# Supplementary material for: Phytochemical Profile and Antioxidant Capacity of Viscum album L. Subsp. album and Effects on Its Host Trees
Source: Plants (Basel). 2022 Nov 9;11(22):3021. doi: 10.3390/plants11223021 (PMC9694764; doi:10.3390/plants11223021)
Supplement: Supplementary file 1 [file plants-11-03021-s001.zip › plants-1941351-supplementary.pdf]

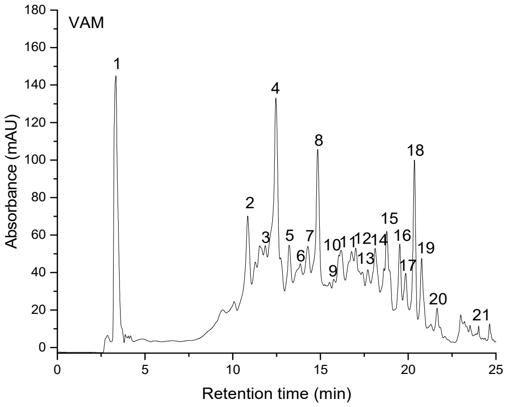

a

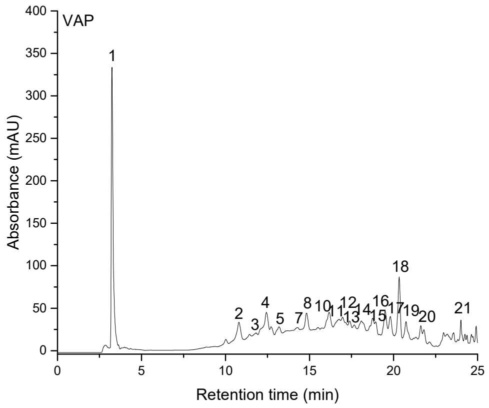

b

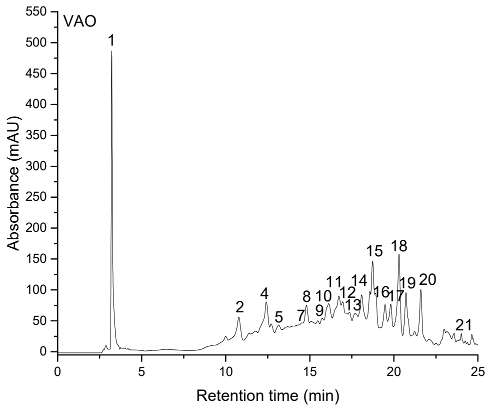

c

Figure S1. HPLC chromatograms of mistletoe leaves according to the host trees. a. VAM (mistletoe grown on *Malus domestica* Barkh.*)*, b. VAP (mistletoe grown on *Prunus domestica* L.), c. VAO (mistletoe grown on *Populus alba* L.)
